# The Influence of Dimethyl Sulfoxide as Electrolyte Additive on Anodic Dissolution of Alkaline Zinc-Air Flow Battery

**DOI:** 10.1038/s41598-019-51412-5

**Published:** 2019-10-18

**Authors:** Soraya Hosseini, Ali Abbasi, Luc-Olivier Uginet, Nicolas Haustraete, Supareak Praserthdam, Tetsu Yonezawa, Soorathep Kheawhom

**Affiliations:** 1 Department of Chemical Engineering, Faculty of Engineering, Chulalongkorn University, Bangkok, 10330 Thailand; 2 Department of Process and Environmental Engineering, INSA Toulouse, Toulouse, France; 3 High-performance computing unit (CECC-HCU), Center of Excellence on Catalysis and Catalytic Reaction Engineering (CECC), Chulalongkorn University, Bangkok, 10333 Thailand; 4 Division of Materials Science and Engineering, Faculty of Engineering, Hokkaido University, Kita 13 Nishi 8, Sapporo, Hokkaido 060-8628 Japan

**Keywords:** Chemical engineering, Batteries, Batteries

## Abstract

The present work describes the effects of dimethyl sulfoxide (DMSO) in KOH aqueous electrolyte on the performance of a zinc-air flow battery. Aqueous electrolytes containing 7 M KOH and (0 to 20)% v/v DMSO were studied revealing a critical role of DMSO on the dissolution and deposition of zinc. The anodic zinc dissolution process was studied via cyclic voltammetry, Tafel polarization and electrochemical impedance spectroscopy (EIS). The presence of DMSO showed improved zinc dissolution performance with the highest peak of zinc dissolution being the electrolyte containing 5% v/v DMSO. Tafel analysis demonstrated a significant decrease in polarization resistance and an increase in corrosion rate due to the introduction of DMSO to the electrolyte. This suggests that DMSO has the ability to suspend zinc oxide in the electrolyte, thus preventing passivation of the zinc surface. EIS results revealed that by adding DMSO to the electrolyte, charge transfer resistance increased. This is attributed to the formation of passive layers having arisen from DMSO adsorption, the formation of zincate ions in the vicinity of the zinc surface, and the deposition of discharged products. A difference in Nyquist plots was observed for 20% v/v DMSO/KOH and 0% v/v DMSO/KOH electrolytes implying non-Debye relaxation behavior taking place due to the surface effects. The electrolytes were implemented in a zinc-air flow battery. Maximum power densities of 130 mW/cm^2^ (5% v/v DMSO) and 125 mW/cm^2^ (20% v/v DMSO) were obtained and were observed to be about 43% and 28% higher than that of the DMSO-free electrolyte. Results indicated that when 20% v/v DMSO was added to KOH solution, there was 67% zinc utilization efficiency (550 mAh/g) which provided 20% improvement in discharge capacity. Further, the battery with 20% v/v DMSO demonstrated excellent cyclability. Overall, DMSO shows great promise for enhancement of zinc dissolution/deposition in zinc-air batteries.

## Introduction

Increasing demands for clean and renewable energy have been widely promoted. This is due to growing energy consumption in modern society enhancing the degree of electrification. However, most renewable energy sources, such as wind and solar, are intermittent. Therefore, great efforts on the development of advanced energy storage technologies have been carried out^1,2^. Various types of batteries are drawing much attention as energy storage devices, considering such characteristics as an abundance of resources, low cost, high energy density, safety and excellent life cycling^3^. Despite the great success of lithium-ion batteries in the market, the limited resources of lithium and their high cost restrict their practical implementation on large-scale application. Therefore, alternative battery technologies which are low in cost and have access to plenty of materials are considered as promising pathways to addressing these limitations^4,5^.

Zinc is a promising alternative owing to its low cost, abundance, inflammability, low toxicity, and promising energy density. Zinc has been extensively used in various types of battery, i.e. zinc-carbon, zinc-alkaline^6^, zinc-nickel^7^, zinc-silver oxide^8^, zinc-ion^9–13^ and zinc-air^14,15^ batteries. Among these, zinc-air batteries exhibit high potential for large-scale application because of their high theoretical specific energy density, which is around 1,084 Wh/kg or five times higher than existing lithium-ion batteries^16^. Although primary zinc-air batteries have been commercialized since the 1930s, several barriers including corrosion, water consumption during cycling, dendrite growth of zinc, electrolyte leakage, zinc oxide precipitation and zincate ion crossover impede the practical application of secondary batteries. These issues are responsible for a decrease in efficiency and poor rechargeable performance^17^. Therefore, many studies have focused on overcoming these issues. Various strategies to suppress dendrite formation such as adding salts, organic and inorganic additives have been proposed^18,19^.

An electrolyte is one of the main factors governing battery electrochemistry. Thus, in order to achieve a long cell performance and improved battery efficiency, selection of a proper electrolyte is essential. The electrochemical behavior of zinc in aqueous solution can be predicted from a Pourbaix diagram^20^. The equilibrium potential between zinc and its various oxidized species depends on the pH of solution. Besides, two possible cathodic reactions, including hydrogen evolution and oxygen reduction, occur in the whole range of pH. Zinc is thermodynamically unstable in aqueous solutions and tends to dissolve along with hydrogen evolution reaction (HER), forming Zn^2+^ ions. Non-faraday HER and zinc corrosion lead to the reduced electrolytic efficiency and life cycle of zinc-air batteries. To overcome the issue, many approaches have been proposed mainly focusing on the use of inorganic additives, organic solvents, and gel polymers^21^. Such additives can affect both the electrode and electrolyte bringing about changes to the crystal growth and structure of zinc deposits.

Various types of metal oxides and metal salts i.e. BaO^22^, Ca(OH)_2_^23^, Al_2_O_3_^24^, Sn_6_O_4_(OH)_4_^25^ and Bi_2_O_3_^18,26^ have been investigated showing several effects: namely, distinct behavior towards zincate formation, inhibiting dendrite growth, suppressing zinc surface passivation, increasing zinc oxide solubility etc. Addition of calcium hydroxide to an electrolyte forms an insoluble compound along with the zincate ions in alkaline electrolytes to maintain uniform distribution leading to improve cyclability^27^. It was found that adding gelling materials to the electrolyte contributes to minimizing water loss and enhancing battery performance and longevity. Othman *et al*.^28^ investigated the viability of hydroponics gel as an additive to a low concentration alkaline solution in a zinc-air primary battery. It was noted that even though hydroponics gel electrolyte was not effective in the electrochemistry of the zinc-air battery, it helped to eliminate battery leakage.

Organic additives in electrolytes have been investigated for suppressing dendritic initiation and propagation in order to prevent a nonuniform zinc deposition. Surfactants (hydrophobic part) are adsorbed on an electrode surface and act as a barrier to prevent access of ions onto the electrode surface. This process leads to higher current densities instead of dendrite formation due to the slow down in deposition rate. Adding sodium dodecyl benzene sulfonate (SDBS) as a surfactant to an alkaline electrolyte effectively hindered surface passivation and improved the discharge capacity of zinc anode^29^. Banik *et al*.^30^ observed that the presence of polyethylene glycol (PEG) in an electrolyte reduced zinc electrodeposition kinetics and suppressed dendrite formation. Further, ethanol, as a green solvent, was examined as an alkaline electrolyte additive to improve the electrochemical performance of a zinc-air flow battery. The electrochemical characteristics demonstrated that the presence of (5–10)% v/v ethanol led to the enhancement of zinc dissolution and prevented zinc anode passivation^31^. The effect of adding two different types of surfactants including sodium dodecyl sulfate (SDS) of 0.2 mM and Pluronic F-127 of 100 ppm to an alkaline electrolyte on zinc-air battery performance was investigated^32^. Results revealed a specific discharge capacity enhancement of 30% and 24% for the electrolytes containing P127 and SDS, respectively.

Dimethyl sulfoxide (DMSO) is known as a green solvent (organosulfur) for inorganic/organic materials having a high dielectric constant and high resistance to redox reactions. Due to very low toxicity, its application has been used in various areas e.g. in medicine (as a pharmaceutical agent), plastic surgery, biological studies, industry (plasticizer), and synthesis of ZnO nanoparticles. Its physicochemical properties indicate that DMSO is highly associated with forming polymer chains due to the interactions between its sulfur and oxygen atoms. However, the effects of temperature or the presence of proton-donor solvents result in self-dissociation of DMSO. The self-dissociation of DMSO probably proceeds accordingly, as in Eq. 1:

1 $$2{({{\rm{CH}}}_{3})}_{2}{\rm{SO}}\rightleftharpoons {({{\rm{CH}}}_{3})}_{2}{{\rm{SOH}}}^{+}+{{\rm{CH}}}_{3}{{\rm{SOCH}}}_{2}^{-}$$

Vakul’skaya *et al*.^33^ studied the combination of KOH/DMSO as a superbase system. It was evident that the physical properties and reactivity of DMSO/KOH resulted in an extraordinary basicity with pKa = 30–32 due to the formation of synergism between two bases, called a superbase media. Several studies have demonstrated the application of DMSO as a solvent to prevent particle sedimentation in various types of solution^34^.

In this work, dimethyl sulfoxide (DMSO) as an organosulfur was selected as an additive to 7 M KOH solution electrolyte in a zinc-air flow battery. The influence of DMSO concentration, ranging from (0 to 20)% v/v, on the sedimentation of ZnO particles generated during battery discharge, was studied. The electrochemical measurements including polarization curve test, cyclic voltammetry (CV), electrochemical impedance spectroscopy (EIS) and chronoamperometry were carried out in order to analyze the electrochemical behavior of DMSO/KOH solution. Furthermore, battery testing was carried out to investigate performance of the battery under various conditions.

## Experimental

### Chemical and Materials

Nickel (Ni) foam, with a purity of 99.97%, 100 pores per inch (PPI) and 1 mm thick, was used as the cathode current collector. It was purchased from Qijing Trading Co., Ltd. 100 mesh of woven wire 304 stainless steel, used as the anode current collector, was purchased from Alikafeii Trading Co., Ltd. Zinc foil with a purity of 99.9% was purchased from Shandong AME Energy Co., Ltd. Zinc granules, with a purity of 99.99% and an average diameter of 0.8 mm, purchased from Sirikul Engineering Ltd., Part., were used as the anode. KOH pellets (99%) and DMSO (99.8%), purchased from CT Chemical Co., Ltd., were used to prepare the electrolytes. Manganese (IV) oxide (MnO_2_, 5 *μ*m 99.99%, Sigma-Aldrich), carbon blacks BP2000 (Cabot Corporation), carbon blacks Vulcan XC-72 (Cabot Corporation), D-glucose (99.5%) and poly(tetrafluoroethylene) (PTFE powder, 1 *μ*m, Sigma-Aldrich) were used to prepare the cathode. Poly (vinyl butyral) (PVB), purchased from Sigma-Aldrich, was used as a binder. Toluene (99.5%) was used as a solvent for the inner layer of the air cathode. Teabag filter paper and poly (vinyl acetate) (PVAc) (TOA Paint Public Co., Ltd.) were used to prepare the separator. All chemicals were used as received and without any further purification.

### Electrode and battery fabrication

**Figure 1:**
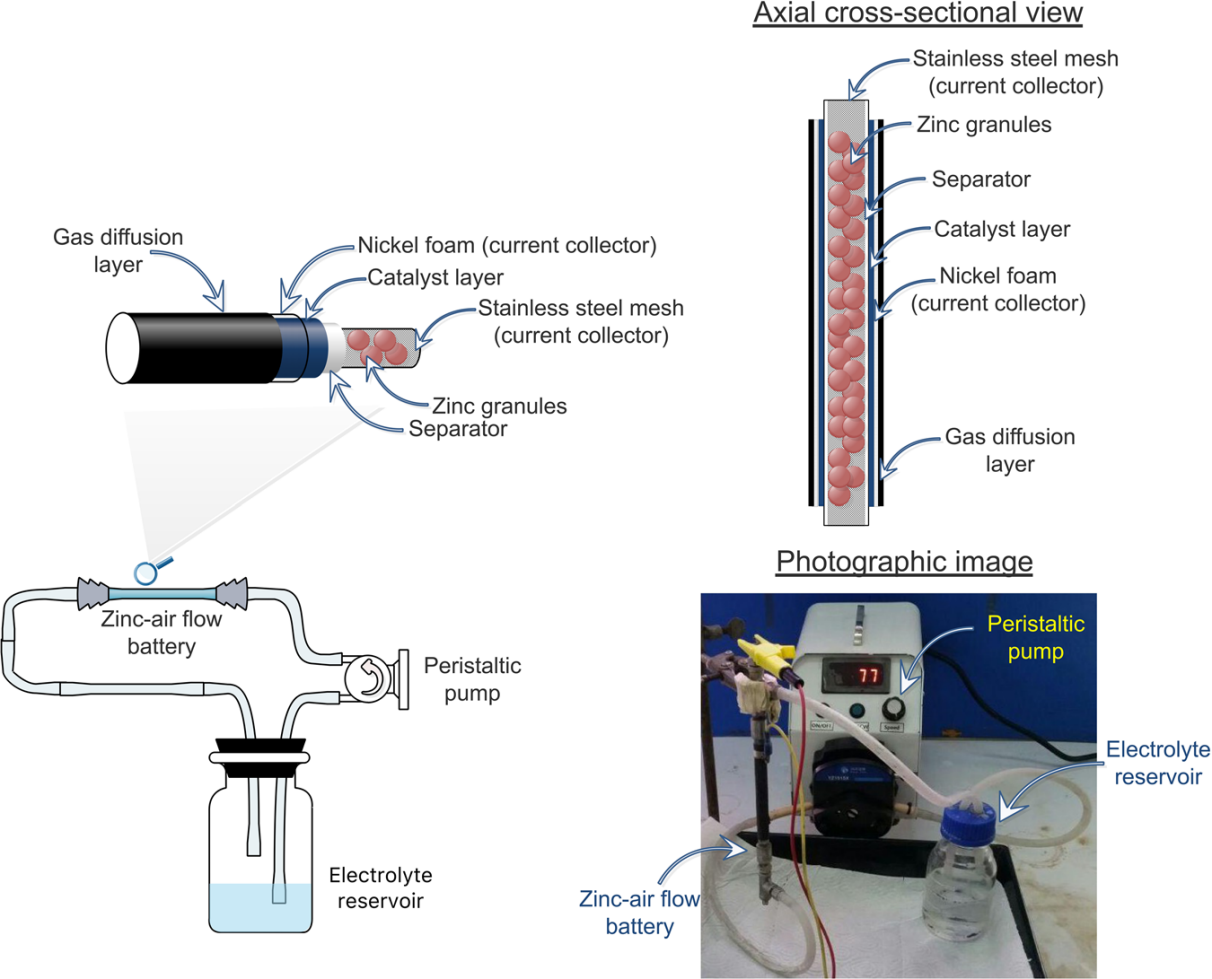
A schematic diagram and photographic image of the zinc-air flow battery.

The air cathode was prepared as a double-side-coated electrode with different compositions. It was fabricated on Ni foam with the inner side (MnO_2_, carbon BP200 and polystyrene-co-butadiene/toluene) as the catalyst and the outer side as the air diffusion layer. As for the air-diffusion layer, the outer surface of the air cathode was prepared by coating a mixture of carbon (XC-72), D-glucose, and PTFE binder in 10 ml ethanol with a ratio of 40:40:20 wt.%, respectively. The well-dispersed paste was applied onto a (5 × 10) cm^2^ nickel foam and then pressed using a manual hot press at 350 °C for 15 min. To prepare the inner side, MnO_2_ (0.3 g) as the catalyst, and carbon BP2000 (0.7 g) were first mixed and dispersed in 8.5 mL of toluene and stirred for 60 min. Then, 1.5 ml of poly (styrene-co-butadiene) in toluene (7.5 wt.%) was added. The mixture was stirred for another 60 min and the prepared catalyst ink was coated two times onto the other side of the previously catalyst-coated Ni foam. Each layer of the coated ink was allowed to fully dry before the subsequent layer was printed. Finally, the coated Ni foam was pressed at 150 °C for 10 min using a manual hot press. Zinc granules were used as the anode packed inside a stainless-steel mesh cylinder (0.5 cm outer diameter). The compact zinc granules were then placed inside the fabricated cell and both sides of the SS mesh were connected to the anode current collector. 7 M KOH solution with various ratios of DMSO was circulated between the anode and separator having a circulating rate of 100 ml/min. The separator was prepared by casting 2 g of 24 wt.% PVAc on a teabag filter paper over both sides and then dried in an oven at 50 °C. The thickness of the dry separator was adjusted to 100 *μ*m using a strip rolling machine at room temperature (25 °C). Next, the separator was cut into pieces, (5 × 11) cm^2^ in size and wrapped around the cell. The air cathode was composed of three layers: the catalyst layer (inner side), the cathode current collector and the gas diffusion layer (outer side). The cathode was wrapped around the separator with the catalyst layer facing inside and tightly fixed in place using cable ties. A schematic view and photographic image of the fabricated cell are shown in Fig. 1.

### Characterization and measurement

Electrochemical techniques comprising of cyclic voltammetry, electrochemical impedance spectroscopy and Tafel experiments were carried out using a potentiostat/galvanostat with impedance measurement unit (AMETEK, PAR VersaSTAT 3A). All electrochemical measurements were performed in 7 M KOH electrolyte. The cell configuration and measurement condition employed in each experiment is described in each corresponding section. Both battery discharge capacity and voltage-current polarization curve were analyzed by battery testing system (NEWARE). The battery system had an electrolyte circulating rate of 100 ml/min throughout at room temperature (25 °C).

## Results and Discussion

### Electrochemical analysis

In Fig. 1, the battery design, separator and electrodes are illustrated. The electrolyte closely relates to the performance of a battery. During discharge of the battery, zinc anode dissolves in the electrolyte to form zincate ions ([Zn(OH)_4_]^2−^), releasing electrons which are then transferred to the cathode through an external circuit. Consequently, saturated zincate ion can precipitate as solid zinc oxide on the active zinc surface. The corresponding reactions are shown in Eqs. 2 and 3.

2 $${\rm{Zn}}+4{{\rm{OH}}}^{-}\to {\rm{Zn}}{({\rm{OH}})}_{4}^{2-}+2{{\rm{e}}}^{-}$$

3 $${\rm{Zn}}{({\rm{OH}})}_{4}^{2-}\leftrightarrow ZnO+2{{\rm{OH}}}^{-}+{{\rm{H}}}_{2}{\rm{O}}$$

Many research works have focused on modifying the electrolyte in order to increase zincate formation and improve battery performance. In this study, DMSO was selected as an additive to 7 M KOH solution to examine zinc dissolution/deposition via electrochemical characterization and their effect on the charge/discharge performance of a zinc-air flow battery.

**Figure 2:**
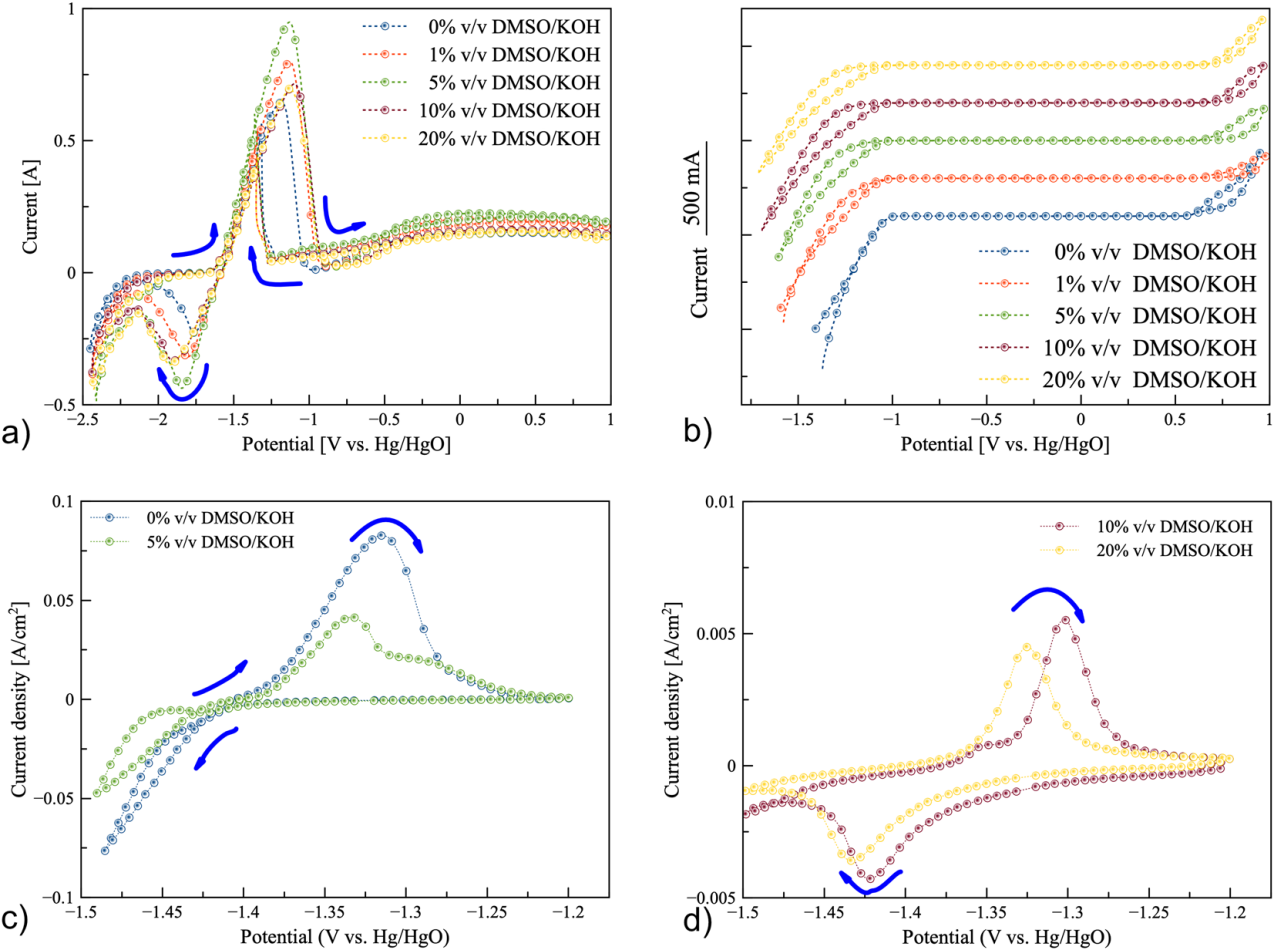
Cyclic voltammograms: (**a**) zinc electrode in (0–20)% v/v DMSO/KOH electrolytes at a scan rate of 0.05 V/s, (**b**) electrochemical windows in (0–20)% v/v DMSO/KOH electrolytes, (**c**) plating/stripping of zinc on Pt electrode in 0 and 5% v/v DMSO/KOH electrolytes at a scan rate of 0.025 V/s, and (**d**) plating/stripping of zinc on a Pt electrode in 10 and 20% v/v DMSO/KOH electrolytes at a scan rate of 0.025 V/s.

Figure 2a shows the cyclic voltammogram of zinc redox at a potential scan rate of 0.05 V/s in 7 M KOH solution containing (0–20)% v/v DMSO/KOH. The tests were carried out in a three-electrode glass cell, having a zinc foil (1 × 1) cm^2^ as working electrode, a platinum (Pt) plate (1 × 1) cm^2^ as counter electrode and Hg/HgO as reference electrode. The potential was initially swept from (−2.5 to 1) V vs. Hg/HgO and then swept back from (1 to −2.5) V vs. Hg/HgO at a potential scan rate of 0.05 mV/s. A prominent anodic peak was attributed to zinc oxidation in the forward scan. The characteristic oxidation peaks occurring between (−1.63 and −0.85) V represented zinc dissolution in the electrolytes.

It was observed that adding various amounts of DMSO increased zincate formation with the highest peak intensity occurring for 5% v/v DMSO/KOH electrolyte. A second anodic peak was also observed in a reverse scan between (−1.27 and −1.55) V vs. Hg/HgO. The backward anodic peak currents varied in proportion to the concentration of DMSO used. On the reverse scan, the zinc deposited is re-oxidized when the critical potential is reached. The critical potential depends on the concentration and aggressiveness of anions. On the forward scan, zinc oxide (ZnO) formation led to its precipitation over the electrode surface and reduced the active area of the electrodes. Second anodic peaks resulted from removal of the zinc oxide precipitated (loose and porous layer) during the forward scan and re-oxidation of the zinc surface. When DMSO was used as an additive to KOH solution, the currents in the anodic dissolution denoted an increase in the electrochemical reaction that may be attributed to the delayed formation of the passivating ZnO layer on the surface of the zinc electrode. Reduction peaks for zinc deposition between (−1.6 to −2.1) V vs. Hg/HgO were observed with maximum current for the 5% v/v DMSO/KOH electrolyte. Further increase of DMSO concentration to (10 and 20)% v/v led to a decrease in the intensity of the current peaks. This could be attributed to an increase in the viscosity of the electrolyte and a decrease in the solubility of zincate in the electrolyte.

The effect of scan rate on the peaks for (0 and 5)% v/v DMSO/KOH electrolytes is shown in the supplementary data (Fig. S1). It was clear that within the potential range of zinc redox reaction, the peak potential separation (ΔE_*p*_) for (0 and 5)% v/v DMSO/KOH electrolytes were 0.67 and 0.73, respectively. Ratios of the cathodic to anodic peak currents (I_*c*_/I_*a*_) for (0 and 5)% v/v DMSO/KOH electrolytes were found to be 0.46 and 0.35, respectively. It was seen that both parameters increased when DMSO was added to KOH solution. Further, the cathodic peaks shifted to a more negative potential while the anodic peaks shifted to a more positive potential. As the scan rate varied, the peak current changed. The change may have originated from the change in the viscosity of the electrolytes affecting zinc ions diffusion as well as the solubility of zinc and consequently, ionic conductivity. CV results showed that the zinc redox reactions were quasi-reversible.

Figure 2b displays the CV results for five electrolytes in order to determine the electrochemical window, at a scan rate of 0.01 mV/s. The tests were carried out in a three-electrode glass cell, having a Pt plate (1 × 1) cm^2^ as working electrode, another Pt plate (1 × 1) cm^2^ as counter electrode and Hg/HgO as reference electrode. The electrolytes containing DMSO revealed a wider electrochemical window compared to the 0% v/v DMSO/KOH electrolyte. Besides, it was found that adding DMSO increased the electrochemical window by 0.12 V.

Figure 2c,d display the CV results for zinc deposition/dissolution on a Pt electrode. The tests were carried out in a three-electrode glass cell, having a Pt plate (1 × 1) cm^2^ as working electrode, a zinc foil (1 × 1) cm^2^ as counter electrode and Hg/HgO as reference electrode. The potential was initially swept from (−1.20 to −1.50) V vs. Hg/HgO and then swept back from (−1.50 to −1.20) V vs. Hg/HgO at a potential scan rate of 0.025 mV/s.

An anodic peak, corresponding to the oxidation of zinc, was detected between (−1.35 to −1.30) V vs. Hg/HgO, for all electrolytes. The current density decreased with increasing content of DMSO from 5% to 20% v/v. Also, the anodic peak shifted negatively as the content of DMSO increased. In the case of 0% and 5% v/v DMSO/KOH electrolytes, zinc deposition did not appear as a current peak, however, the current increased continuously indicating that no mass-transfer-limited current for zinc deposition and HER. In comparison, 10% and 20% v/v DMSO/KOH electrolytes revealed the cathodic peak around −1.42 V vs. Hg/HgO. It is evident that increasing of DMSO content decreased the ionic conductivity along with suppressing HER. Also, the significant HER was observed only in 0% and 5% v/v DMSO electrolytes.

Corrosion behavior of zinc anode can be investigated via Tafel experiments. Then, the polarization method can be used for obtaining the corrosion parameters. The zinc anode was scanned over a range of potential in the electrolytes. The relation between potential and logarithm of current density can be expressed, as given in Eq. 4:

4 $$\eta =\frac{2.3RT}{\alpha F}\,\log \,i-\frac{2.3RT}{\alpha F}\,\log \,{i}_{0}$$

where *i* is the reaction current density. *i*_0_ is the exchange current density. *F* is the Faraday constant. *R* is the gas constant. *T* is the absolute temperature. *α* is the symmetry factor. The two parameters of corrosion potential (*E*_corr_) and corrosion current density (*I*_corr_) can be determined by extrapolating linear regions of the cathodic and anodic curves. The anodic and cathodic Tafel constants *α*_*a*_ and *α*_*c*_ are the slopes of the overvoltage curves that can be estimated. These parameters can be used to determine the corrosion rate for linear polarization resistance, defined as in Eq. 5:

5 $${\rm{c}}{\rm{o}}{\rm{r}}{\rm{r}}{\rm{o}}{\rm{s}}{\rm{i}}{\rm{o}}{\rm{n}}\,{\rm{r}}{\rm{a}}{\rm{t}}{\rm{e}}=\frac{0.13{I}_{{\rm{c}}{\rm{o}}{\rm{r}}{\rm{r}}}{\rm{E}}{\rm{W}}}{d}$$

where *d* is the sample density and EW is the equivalent weight of the sample. By assuming a small over potential, the anodic and cathodic Tafel constants together with the polarization resistance (*R*_*p*_) can be estimated as in Eq. 6:

6 $${R}_{p}=\frac{{\alpha }_{a}{\alpha }_{c}}{2.33{I}_{{\rm{corr}}}({\alpha }_{a}+{\alpha }_{c})}$$

**Figure 3:**
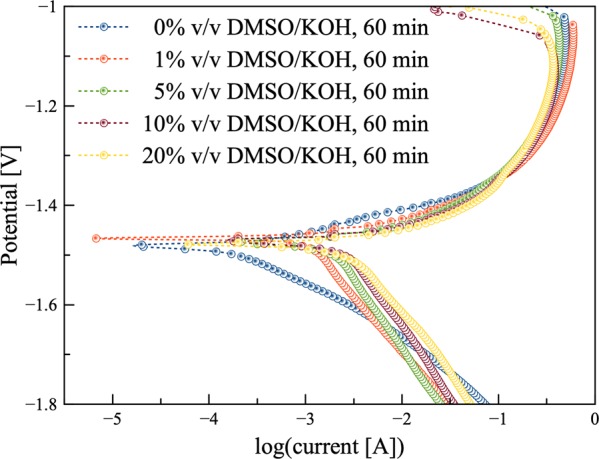
Potentiodynamic polarization measurement of the zinc anode at 60 min after soaking using 0.16 mV/s within the potential range of −0.3 V to 0.3 V vs. OCV.

Herein, Tafel experiments were carried out at 0.16 mV/s within the potential range of (−0.3 to +0.3) V vs. open circuit voltage (OCV). Figure 3 shows the Tafel plots (potential vs. logarithm of the current density) of the zinc anode in various electrolytes at 60 min soaking time. In Table 1, the corrosion potential (*E*_corr_) and corrosion current density (*I*_corr_) and other parameters are listed. The presence of DMSO results in a slight shifting in both cathodic and anodic branches of the Tafel plots, suggesting that DMSO affects both cathodic and anodic reactions. Fig. S2 shows the Tafel plots for zero soaking time; the currents of the anodic part exhibit a similar trend for all electrolytes with passivation potential between (−1.0 and −1.2) V. When 0% v/v DMSO/KOH electrolyte was used, the currents of the cathodic part slightly declined. When DMSO content increased, the turning points of the currents slightly changed. When DMSO content increased from (0 to 20)% v/v, the corrosion potential shifted negatively from (−1.49 to −1.47) V. The electrolyte containing 20% v/v DMSO/KOH showed the highest *I*_corr_ while 0% v/v DMSO/KOH electrolyte exhibited the minimum *I*_corr_. The electrode potential at open-circuit was found to be −1.48 V vs. Hg/HgO for 7 M KOH solution after 60 min soaking. This was due to the oxidation of zinc, the reduction of water and ZnO deposition (Fig. 3).

**Table 1:** Parameters of Tafel analysis for the zinc anode in electrolytes containing DMSO from (0 to 20)% v/v.

Electrolyte	*E*_corr_ (V)	log(*I*_corr_) (A)	*α*_*a*_	*α*_*c*_	*R*_*p*_ (Ω)	corrosion rate
**0 min soaking time**						
0% v/v DMSO/KOH	−1.49	−3.3	0.752	0.188	0.130	0.298
1% v/v DMSO/KOH	−1.48	−2.6	0.501	0.158	0.021	1.495
5% v/v DMSO/KOH	−1.48	−2.7	0.501	0.116	0.020	1.188
10% v/v DMSO/KOH	−1.48	−2.6	0.601	0.111	0.016	1.495
20% v/v DMSO/KOH	−1.47	−2.4	0.601	0.107	0.010	2.307
**60 min soaking time**						
0% v/v DMSO/KOH	−1.48	−3.50	0.601	0.231	0.229	0.188
1% v/v DMSO/KOH	−1.47	−3.00	0.752	0.131	0.048	0.595
5% v/v DMSO/KOH	−1.47	−2.75	0.601	0.107	0.022	1.058
10% v/v DMSO/KOH	−1.47	−2.60	0.501	0.111	0.016	1.495
20% v/v DMSO/KOH	−1.47	−2.30	0.429	0.097	0.007	2.983

Compared with the DMSO-free electrolyte, the electrolytes having different ratios of DMSO, from (1 to 20)% v/v showed higher corrosion potentials and currents. In general, the potential is a thermodynamic value while the current is a kinetic value, depending on various parameters. Corrosion rate is estimated from the corrosion current density along with the surface area of the working electrode (zinc); corrosion rate changes due to porosity and accumulation of corrosion products. Indeed, the surface area of a working electrode affects corrosion current density. The surface area was estimated using chronoamperometry method. Results indicated that the surface area increased from (2 to 3.15) cm^2^ after 60 min soaking when 0% v/v DMSO/KOH electrolyte was used. This was due to the generated ZnO covering the electrode surface (porous layer). When DMSO was added, the surface area was expected to increase to more than 3 cm^2^. However, the final surface area was found to be smaller than 3 cm^2^, indicating that less amount of ZnO accumulated over the surface. Therefore, the zinc surface was more exposed to the electrolyte (DMSO/KOH) facilitating the diffusion of hydroxide groups. It was observed that the counter electrode (Pt plate) was coated with a white precipitate for the electrolyte containing DMSO which increased when the amount of DMSO increased. The white precipitate was analyzed via EDX technique. Results revealed that it contained 34.17 wt.% O and 65.83 wt.% Zn. Thus, it was confirmed that ZnO was suspended and coated over the platinum plate. It can be concluded that a repulsion force may occur between DMSO and ZnO in which the generated ZnO is not able to deposit over the zinc surface. As a result of suspending ZnO from the working electrode surface, there was excellent dispersion of ZnO nanoparticles in DMSO. For this reason, when DMSO ratio increased, the dissolution rate increased.

When DMSO ratio increased from (0 to 20)% v/v, *E*_corr_ increased from (−1.48 to −1.47) V. The cathodic curves showed similar trends for electrolytes with and without DMSO. Furthermore, after a sharp increase in corrosion potential, a similar trend was observed for the anodic curves of all electrolytes with a breakpoint of −1.15 V. This indicated that a thin protective layer forms after reaching passivation potential *E*_pp_. The effect of DMSO as regards anodic reaction was more significant compared to its effect on the cathodic reaction; a shifting of the corrosion potential towards the anodic direction appeared. The low *I*_corr_ indicated the weakening activity of the ion, and the enhancing of corrosion resistance. Besides, when DMSO concentration increased, there was no significant difference in the Tafel slopes (*α*_*a*_, *α*_*c*_) indicating that the presence of DMSO in the electrolyte does not mocisms^35^. Polarization resistance (*R*_*p*_) is the transition resistance between the electrodes and the electrolyte. Thus, *R*_*p*_ increased from (0.13 to 0.3) Ω for KOH solution while for 20% v/v DMSO/KOH a decrease from (0.01 to 0.007) Ω was observed. Estimated values from Tafel constants demonstrated a decrease in the resistance value from (0.3 to 0.007) Ω when DMSO concentration increased from (0 to 20)% v/v during 60 min soaking. Results confirmed that DMSO caused a decrease in the electrode/electrolyte interface resistance except for 1% v/v DMSO/KOH. This demonstrated an increase in the resistance from (0.021 to 0.048) Ω. Corrosion rate increased when DMSO concentration increased, due to the ability of the DMSO/KOH for suspending and dissolving the oxide surface film, resulting from zinc dissolution^36^.

**Figure 4:**
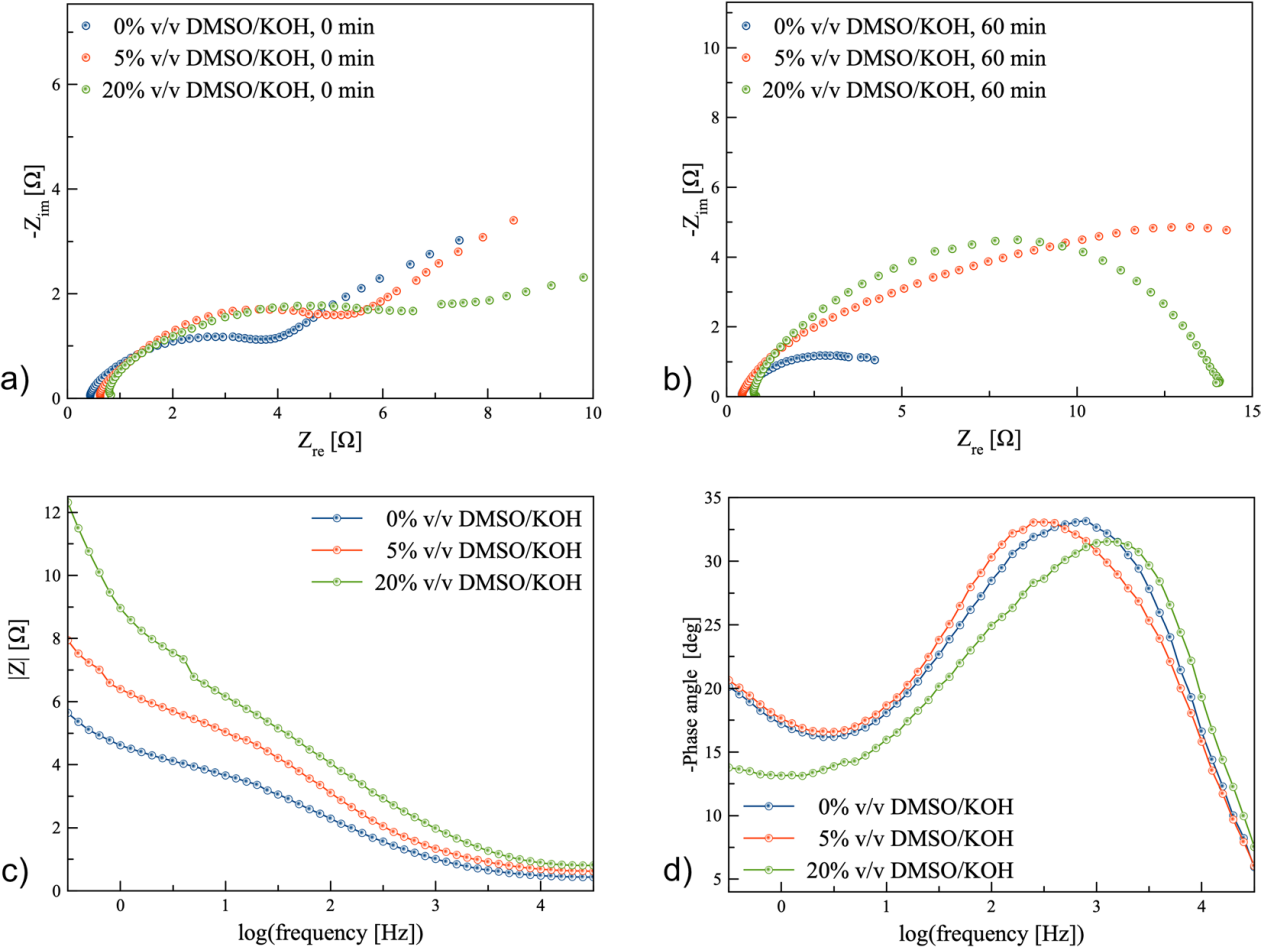
Electrochemical Impedance spectroscopy performed at the potential 0.0 V (vs. OCV) in the frequency range 100 kHz to 0.01 Hz with alternate current amplitude of 10 mV: (**a**) Nyquist plot of 0%, 5%, and 20% DMSO/KOH electrolyte immediately after soaking, (**b**) Nyquist plot of 0%, 5%, and 20% DMSO/KOH electrolyte at 60 min after soaking, (**c**) Bode plot of log|Z| vs. log(*f* ), and (**d**) log(*f* ).

EIS technique was carried out to examine various electrolytes containing (0 to 20)% v/v DMSO/KOH. The measurements were performed at a frequency range from 100 kHz to 0.01 Hz with alternate current (AC) amplitude of 10 mV around OCV. In Fig. 4a,b, the corresponding Nyquist plots are shown. Each spectrum consisted of one semicircle in the high-frequency region followed by one straight line (Warburg) in the low-frequency area. Warburg impedance resulted from the reaction at the passivated zinc surface because of the accumulation of oxide/hydroxide layers^37^. EIS results also showed that the diameter of the semicircle increased when DMSO was added to the electrolyte due to the formation of a passive layer of DMSO over the zinc surface. The Nyquist plots were recorded during (0 to 60) min for all electrolytes, as shown in the supplementary data (Figs S3–S5). The Nyquist plot for 0% v/v DMSO/KOH electrolyte showed an increase in the diameter of the semicircle from (0 to 60) min due to the accumulation of oxidation products. However, the Nyquist plots of the electrolyte containing 20% v/v DMSO were significantly depressed without the straight line, and the semicircle diameter decreased (after 30 min) indicating a wide frequency dispersion of the double layer capacitance involved in the oxidation of zinc.

R_*s*_ refers to contact resistance. Interface resistance was assigned as the electrolyte and solid interface resistance. Charge transfer resistance was associated with the charge transfer at the electrode during the electrochemical reactions. Interface resistance together with the charge transfer resistance significantly varied due to the addition of DMSO. The formation and precipitation of ZnO as an insulating layer had an effect on the electrolyte/electrode interface along with interaction/repulsion between DMSO and ZnO. The electrolytes containing (5 to 20)% v/v DMSO had a relatively larger impedance than that of the electrolyte containing 0% v/v DMSO (0 min) due to the presence of a passive layer of DMSO. However, after 60 min significant differences were observed between the electrolytes (with and without DMSO). These results demonstrated that the introduction of DMSO may attribute to an improvement in the wettability of the zinc surface as well as increasing electrolyte conductivity. On the electrode surface that is wetted by the electrolyte and more wetted surface, an electric double layer is located. This resulted in an increase in the double layer capacitance; as a consequence, ionic conductivity increased^38^. The higher roughness of the zinc surface due to the precipitation of oxidation products led to a larger surface area, reducing ohmic resistance and the metallic gloss of the zinc surface disappeared. It was reported that the presence of additives affected the roughness and glossy metal texture and therefore, its conductivity. The films generated over the zinc surface prevented the zinc from further oxidation and increased the resistance. After 30 min, the damage of the passive layer to improve OH^−^ diffusion reduced precipitation of zinc oxide and further suspension of ZnO due to the presence of DMSO which caused a decrease in resistance from (18 to 13) Ω.

Impedance results were fitted using an equivalent circuit model consisting of various elements. In Table 2, impedance parameters obtained from the fitting models are listed. Due to enhanced electrolyte components, R_*s*_ values increased from (0.43 to 0.81) Ω and DMSO concentration increased from (0 to 20)% v/v. When DMSO concentration increased, the slope of the lines decreased and the lines bend towards the real (Z_re_) axis suggesting the relaxation to be non-Debye type due to the surface effects^39,40^. A difference was observed for the fitted models between (0 and 20)% v/v DMSO/KOH electrolytes. This could be assigned to the formation and accumulation of zincate ions near the surface of the zinc electrode. Zincate ions can diffuse into the bulk solution and can also form a network near the electrode surface. Indeed, the possible mechanisms of zinc passivation include the presence of additives, a zinc oxide layer and the formation of a network of zincate ions in the vicinity of zinc anode. Zincate ions may form hydrogen bonds with DMSO, leading to the enhancement of the network in the vicinity of the electrode surface (porous structure). EIS results showed higher resistance when DMSO covered the electrode but the porous structure of the passive layer allowed the diffusion of hydroxide ions to the electrode surface^41^. Resistances R_1_, R_2_ and R_3_ remarkably increased with the addition of DMSO. This may be due to the formation of a network of zincate ions in the vicinity of zinc and bonding with DMSO.

**Table 2:** The obtained parameters from fitting of EIS data.

	0%v/v DMSO/KOH	5%v/v DMSO/KOH	20%v/v DMSO/KOH
R_*s*_(Ω)	0.4391	0.4721	0.8104
C (F)	1.339 × 10^−4^	2.175 × 10^−4^	1.527 × 10^−4^
R_1_(Ω)	0.9202	2.135	3.117
C (F)	6.249 × 10^−4^	12.450 × 10^−4^	0.760 × 10^−4^
R_2_(Ω)	1.673	5.020	9.565
C (F)	8.803 × 10^−4^	2.574 × 10^−1^	
R_3_(Ω)	1.740	8.581	

Figure 4c,d exhibit Bode plots of the electrolytes containing 0%, 5%, and 20% v/v DMSO/KOH. It was noted that the |Z| value decreased when the frequency increased, suggesting the behavior can be considered as a diffusion-controlled reaction. The Bode plots of 0%, 5%, and 20% v/v DMSO/KOH electrolytes demonstrated a non-straight line. At low frequency, highest resistance was observed for 20% v/v DMSO/KOH electrolyte. Thus, this suggests that a network from DMSO and ZnO can be formed leading to an increase in resistance and better protection performance. In comparison, 0% v/v DMSO/KOH electrolyte indicated the lowest resistance due to the formation of ZnO film. Low-frequency impedance rose sharply for 20% v/v DMSO/KOH electrolyte compared to other electrolytes which may be attributed to surface passivation by a high concentration of DMSO. The phase plots (Fig. 4d) demonstrate capacitive regions at intermediate frequencies with two overlapped phase maxima at intermediate and low frequencies. Zinc electrode has a specific time constant that determines the frequency range that a peak (inflection) appears in the Bode plot; the fastest process occurs at high frequency. Further, the peak shifted towards high frequency when the electrolyte containing 20% v/v DMSO/KOH was applied.

The enhancement of zinc dissolution can be attributed due to the formation of a passive layer in which EIS proved the increase in total resistance. Indeed, DMSO acted as an adsorbent onto the zinc surface with excellent wetting properties leading to an increase in zinc dissolution along with controlling ZnO deposition due to decreasing resistance after long-time soaking. It can be concluded therefore that DMSO molecules adsorb onto the zinc surface to prevent ZnO deposition and also contribute to the suspension of ZnO in the bulk electrolyte.

### Discharge performances

**Figure 5:**
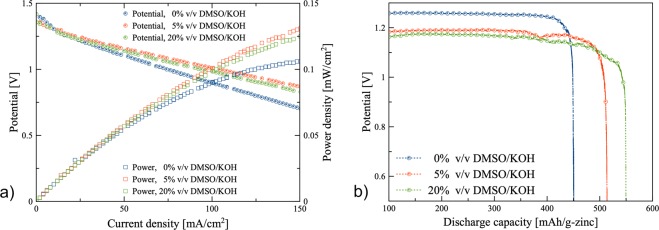
Performance of the zinc-air flow batteries: (**a**) polarization characteristics of the batteries using different electrolytes with the electrolyte circulation rate of 100 mL/min, and (**b**) galvanostatic discharge profiles of the batteries using different electrolytes with the electrolyte circulation rate of 100 mL/min.

Electrochemical performances of zinc-air batteries using electrolytes containing 0%, 5%, and 20% v/v DMSO/KOH were examined employing zinc-air flow batteries operated at an electrolyte circulation rate of 100 mL/min. As shown in Fig. 5a, the power and voltage of the batteries are functions of the discharged current density. The discharge characteristics, in all cases, are almost similar. The batteries exhibited an open circuit potential around 1.40 V. All cases showed a decrease in discharge voltage with an increase in discharge current density. At discharge current density above 10 mA/cm^2^, a linear drop of voltage was observed indicating that ohmic losses dominated cell performance. At current density of 150 mA/cm^2^, the cells with the electrolytes containing (0, 5, and 20)% v/v DMSO/KOH showed (0.71, 0.87, and 0.83) V, respectively. Besides, at 150 mA/cm^2^, the power density of the batteries using electrolytes containing 5% and 20% v/v DMSO/KOH were 130 mW/cm^2^ and 125 mW/cm^2^ which are about 43% and 28% higher than that of 0% v/v DMSO/KOH electrolyte.

Results indicated that when DMSO was added into the KOH-based electrolyte, it had a positive effect on the performance of the battery. In Fig. 5b, galvanostatic discharge profiles of the batteries using different electrolytes are shown. Thus, the battery using 0% v/v DMSO/KOH electrolyte had an initial discharge voltage of 1.26 V which was greater than that of the batteries using 5% v/v DMSO/KOH electrolyte (1.19 V) and 20% v/v DMSO/KOH electrolyte (1.17 V). The voltage profiles of 0%, 5%, and 20% v/v DMSO/KOH electrolytes demonstrated similar trends. The battery with higher DMSO concentration offered greater capacity with higher overpotential compared to other electrolytes due to decreased ionic conductivity of the electrolyte. The specific capacities obtained were (450, 515, and 550) mAh/g_Zn_ for (0, 5, and 20)% v/v DMSO/KOH electrolytes, respectively. The battery with 20% v/v DMSO/KOH electrolyte provided an active material utilization of 67% according to the value of 820 mAh/g_Zn_ for the theoretical zinc-specific capacity. Therefore, the material utilization of zinc granular with the electrolyte containing 20% v/v DMSO was 20% higher than that of the DMSO-free electrolyte at 10 mA/cm^2^ discharge current density. The increase can be attributed to the partial formation of a passive layer on the Zn anode surface limiting its utilization during discharging and suspending zinc oxide particles from the surface of the zinc anode.

**Figure 6:**
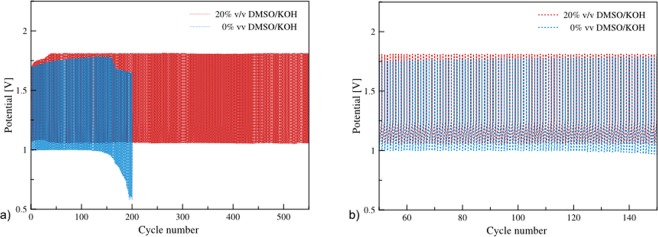
Galvanostatic charge/discharge profiles of the batteries using different electrolytes with the electrolyte circulation rate of 100c/min and repeated cycling discharge 75 mA/cm^2^ for 5 mAh and charge 25 mA/cm^2^ for 5 mAh: (**a**) during 1th-600th cycle, and (**b**) during 50^th^–150^th^ cycle.

Charge/discharge performance of the batteries using electrolytes containing 0%, and 20% v/v DMSO/KOH were examined at an electrolyte circulation rate of 100 mL/min. In each case, 0.5 M ZnO was added initially to the electrolytes. The batteries were discharged at 75 mA/cm^2^ for 5 mAh and followed by charging at 25 mA/cm^2^ for 5 mAh. The charge/discharge was performed cyclically for 600 cycles or until the battery died. As shown in Fig. 6, the galvanostatic charge/discharge characteristics of the batteries are displayed. At initial cycles, the battery using 0% v/v DMSO/KOH electrolyte exhibited discharge voltage at 1.0 V, which was lower than the discharge voltage offered by 20% v/v DMSO/KOH electrolyte. Moreover, the discharge voltage dropped as the cycle increased. The significant degradation was observed after 150^th^ cycle. Finally, the battery died at 198^th^ cycle. In comparison, the battery using 20% v/v DMSO/KOH electrolyte exhibited stable discharge voltage at 1.1 V. Nevertheless, the charge voltage was 1.85 V, which was greater than that of 0% v/v DMSO/KOH electrolyte. Results revealed that the battery using 20% v/v DMSO/KOH electrolyte was highly stable and can be cycled more than 600 cycles without significant performance loss.

**Figure 7:**
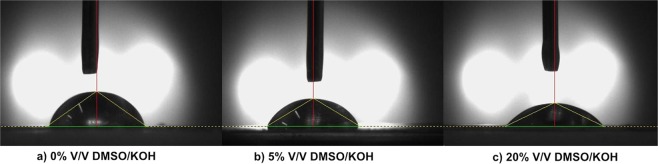
Results of sessile drop test of different electrolytes containing 0%, 5%, 20% v/v DMSO/KOH electrolytes on the zinc surface.

Sessile drop technique was adopted to investigate the wetting behavior of the electrolytes on the zinc surface (1 × 4 m^2^) using CAM-Plus Image machine. In Fig. 7, the sessile drops of different electrolytes with the contact angles are shown. Poor wettability and adhesion can be obtained for a contact angle greater than 90° and less than 180° while high wettability with good adhesion is demonstrated by a contact angle less than 90°. Surface tension and viscosity define electrolyte wettability. Equilibrium contact angle, surface roughness and shape of a boundary are the parameters that affect wetting properties. Detailed pictures of each drop of 0%, 5%, and 20% v/v DMSO/KOH electrolyte are 70.24°, 61.93°, and 51.22°, respectively. The electrolyte with 0% v/v DMSO/KOH showed poor wettability compared to the electrolyte containing 20% v/v DMSO which improved by 27% and the electrolyte spread easily over the zinc surface.

Results revealed that DMSO effectively enhanced performances of zinc-air flow batteries. Other studies reported the effects of different additives in zinc-based batteries^42,43^. In a zinc-nickel flow battery, tetraethylammonium hydroxide, tetraethylammonium bromide and polyethylene glycol 200 were reported as promising additives providing about 10% enhancement in energy efficiency^43^. Calcium oxide was implemented as an additive in a zinc-air flow battery^42^. Improved columbic efficiency was reported. The presence of metal particles as a nucleus for zinc deposition. Importantly, adding an additive to electrolyte causes an improvement in battery efficiency due to regular zinc deposition during charging process. Herein, enhancement in discharge capacity and excellent cyclability of a zinc-air flow battery was achieved using DMSO additives in aqueous KOH electrolyte.

## Conclusion

The aim of this work is to study the potential electrolyte additive DMSO in a zinc-air flow battery. DMSO as an organosulfur compound indicates an ability for producing colloidal solutions to the synthesis of ZnO nanoparticles. For this reason, DMSO in 7 M KOH presents a useful strategy for suspending generated ZnO in order to prevent passivation as well as to improve electrochemical performance and discharge capacity. Electrochemical characterization revealed that an increase of 41% and 0.12 V was observed in the anode dissolution and electrochemical window, respectively. Besides, battery testing showed that DMSO significantly enhanced discharge capacity by 20% and demonstrated excellent cyclability. Hence, due to this environmentally friendly green solvent and its effectiveness in increasing battery performance, the utilization of DMSO can be considered a promising additive for zinc-air batteries.

## Supplementary information

Supplementary Information

## Data Availability

The authors declare that all relevant data are within the paper.
