# Supplementary material for: The Influence of Dimethyl Sulfoxide as Electrolyte Additive on Anodic Dissolution of Alkaline Zinc-Air Flow Battery
Source: Sci Rep. 2019 Oct 18;9:14958. doi: 10.1038/s41598-019-51412-5 (PMC6802117; doi:10.1038/s41598-019-51412-5)
Supplement: Supplementary file 1 — Supplementary Information [file 41598_2019_51412_MOESM1_ESM.docx]

**The Influence of Dimethyl Sulfoxide as Electrolyte Additive on Anodic Dissolution of Alkaline Zinc-Air Flow Battery**

Soraya Hosseini^1^, Ali Abbasi^1^, Luc-Olivier Uginet^1,2^, Nicolas Haustraete^1,2^, Supareak Praserthdam^1,3^, Tetsu Yonezawa^4^, and Soorathep Kheawhom^1,*^

^1^Department of Chemical Engineering, Faculty of Engineering, Chulalongkorn University, Bangkok, 10330, Thailand

^2^Department of Process and Environmental Engineering, INSA Toulouse, France

^3^High-performance computing unit (CECC-HCU), Center of Excellence on Catalysis and Catalytic Reaction Engineering (CECC), Chulalongkorn University, Bangkok, 10333, Thailand

^4^Division of Materials Science and Engineering, Faculty of Engineering, Hokkaido University, Kita 13 Nishi 8, Sapporo, Hokkaido, 060-8628, Japan

*soorathep.k@chula.ac.th

**Supplementary Information**


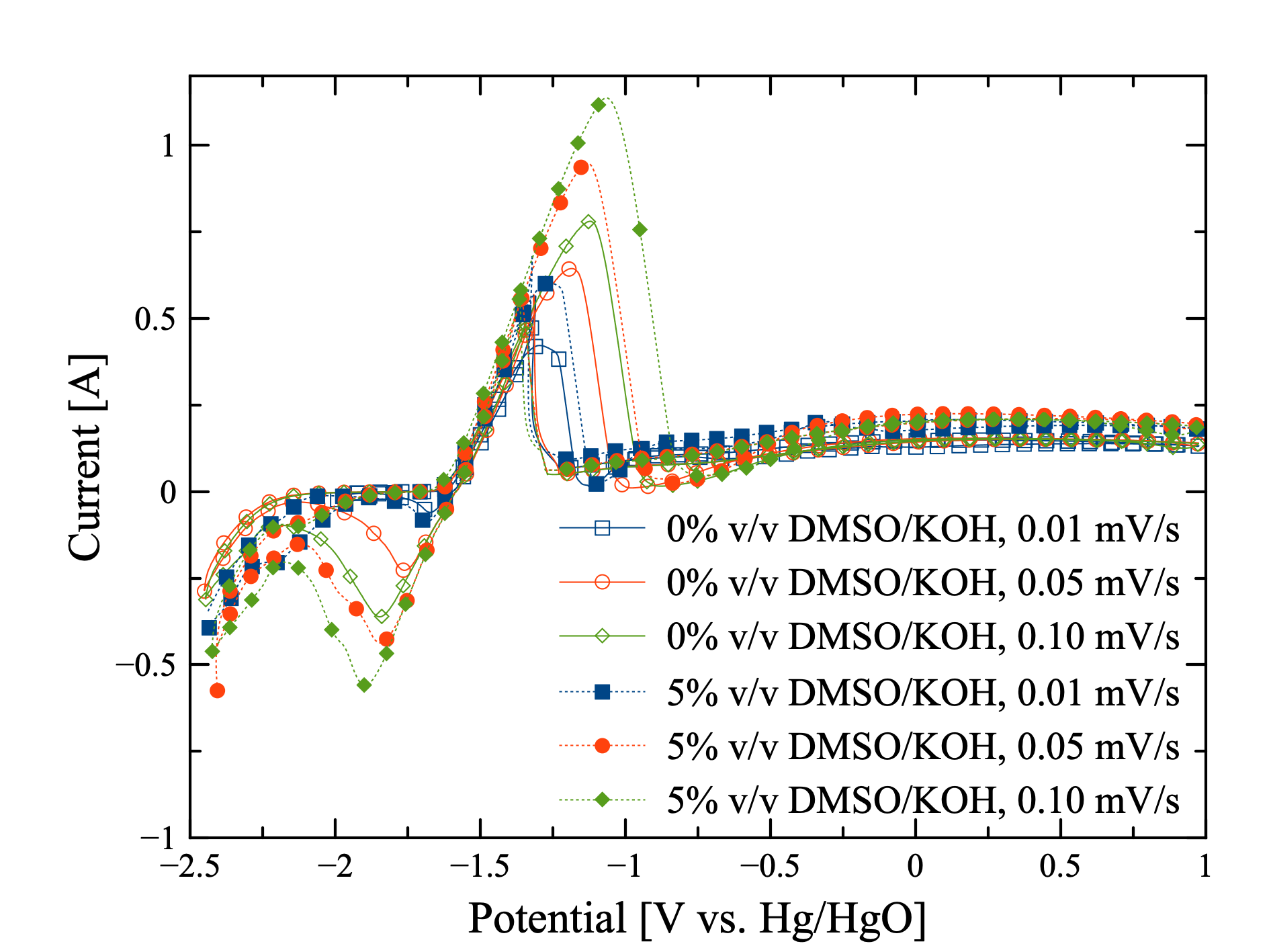


**Figure S1.** Cyclic voltammograms of zinc electrode in 0% and 5% v/v DMSO/KOH electrolytes at different scan rates.


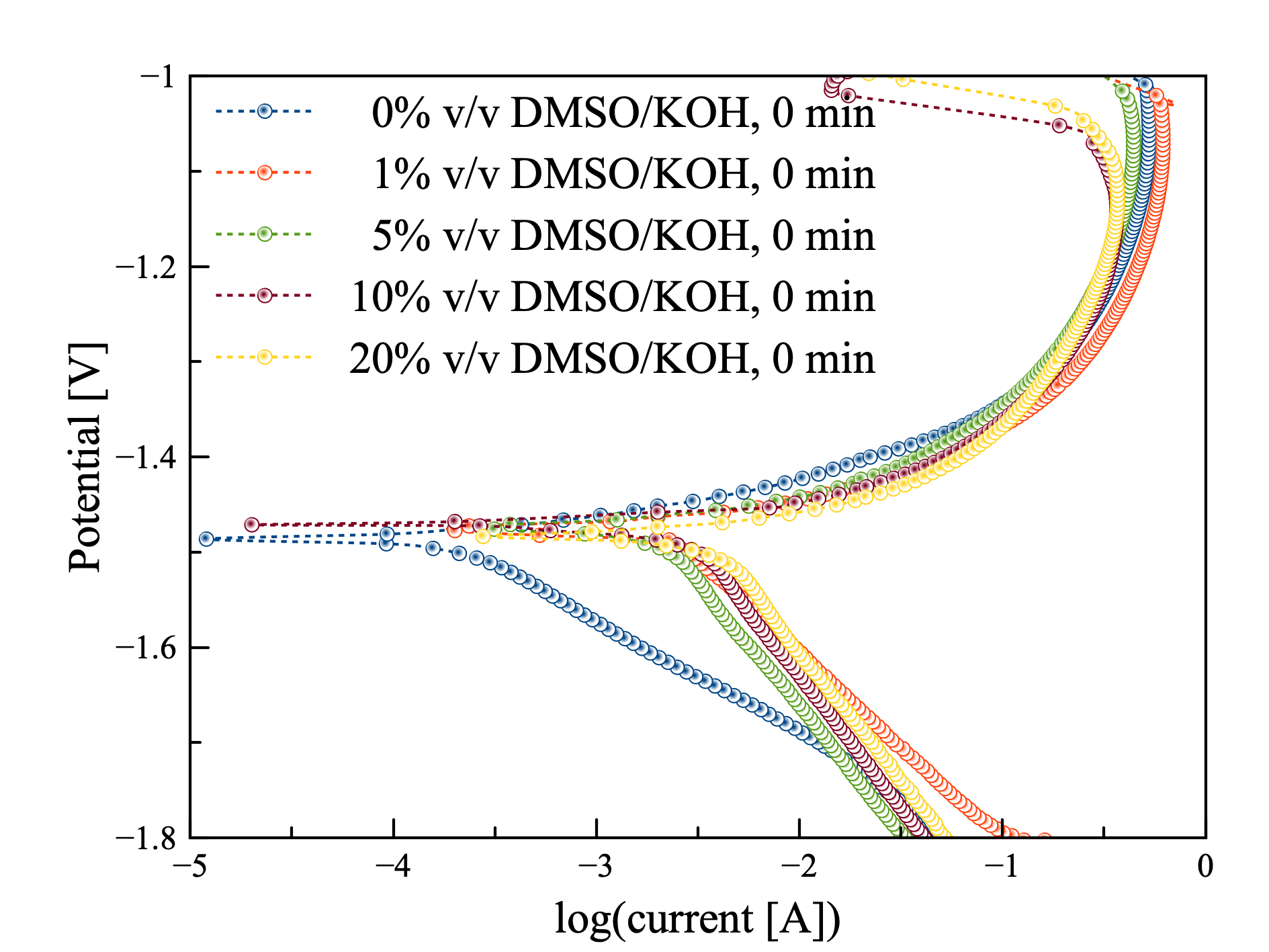


**Figure S2.** Potentiodynamic polarization measurement of the zinc anode immediately after soaking using 0.16 mV/s within the potential range of -0.3 V to 0.3 V vs. OCV.


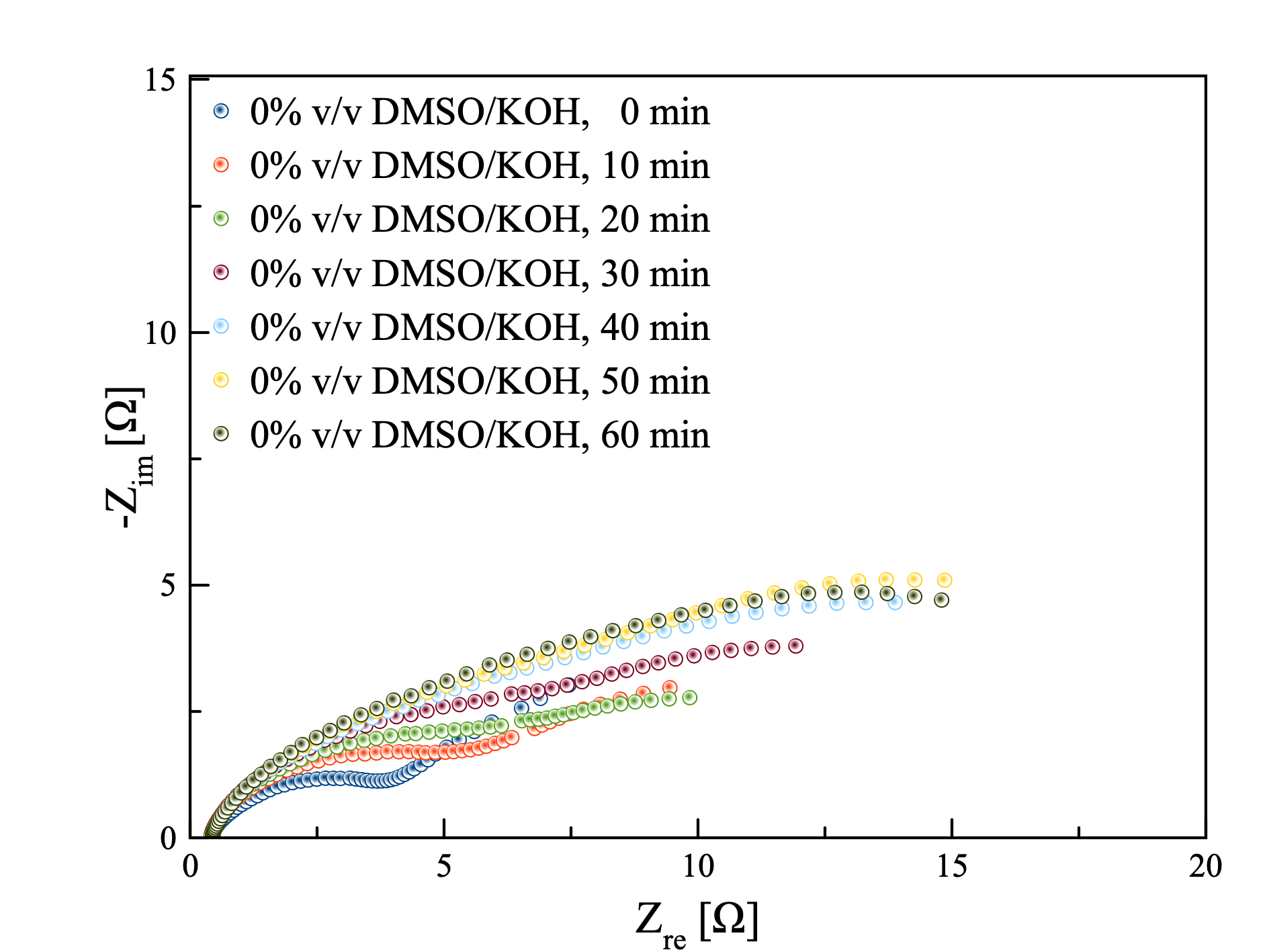


**Figure S3.** Nyquist plot of 0% v/v DMSO/KOH electrolyte at different soaking times (0-60 min).


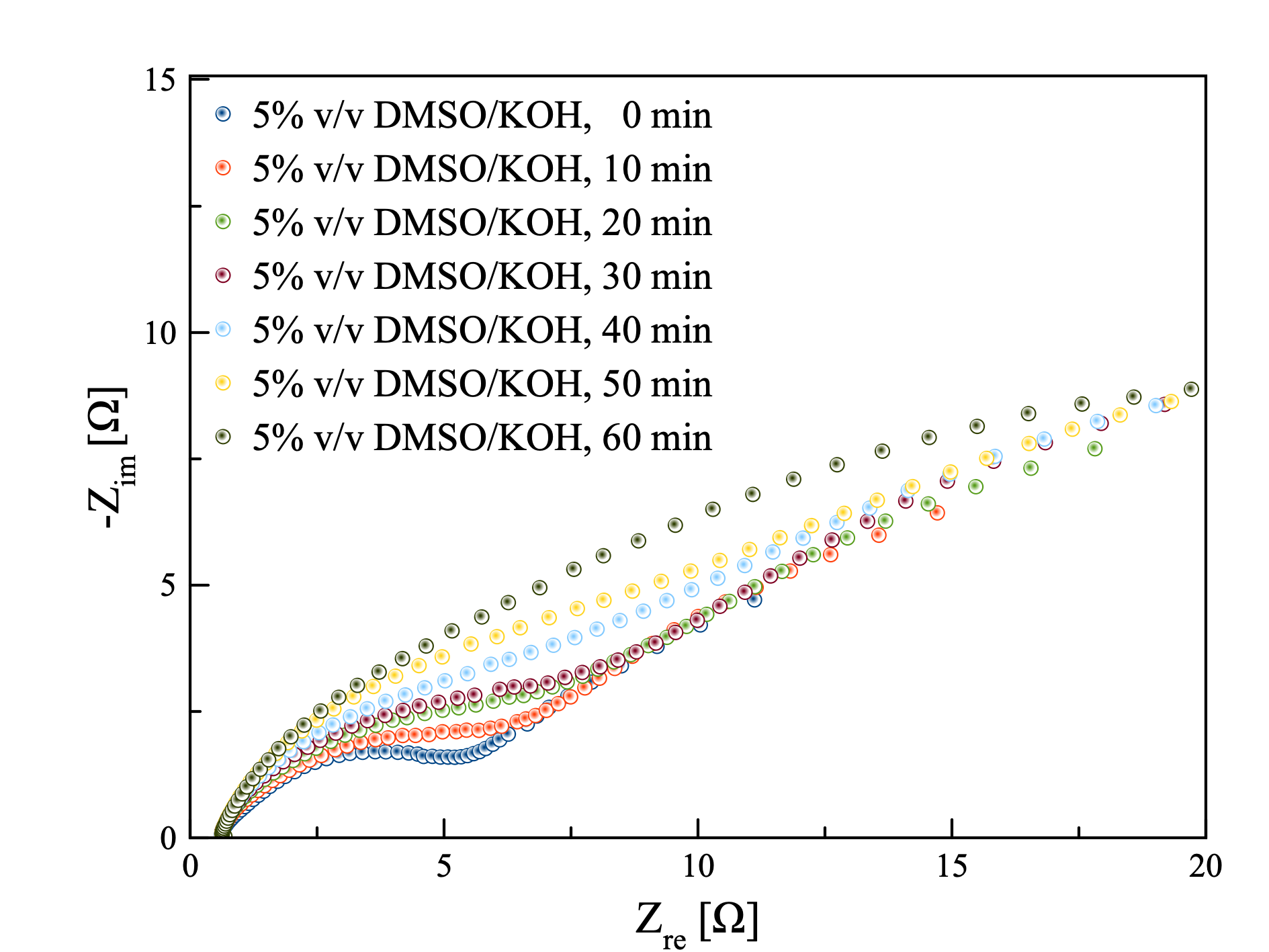


**Figure S4.** Nyquist plot of 5% v/v DMSO/KOH electrolyte at different soaking times (0-60 min).


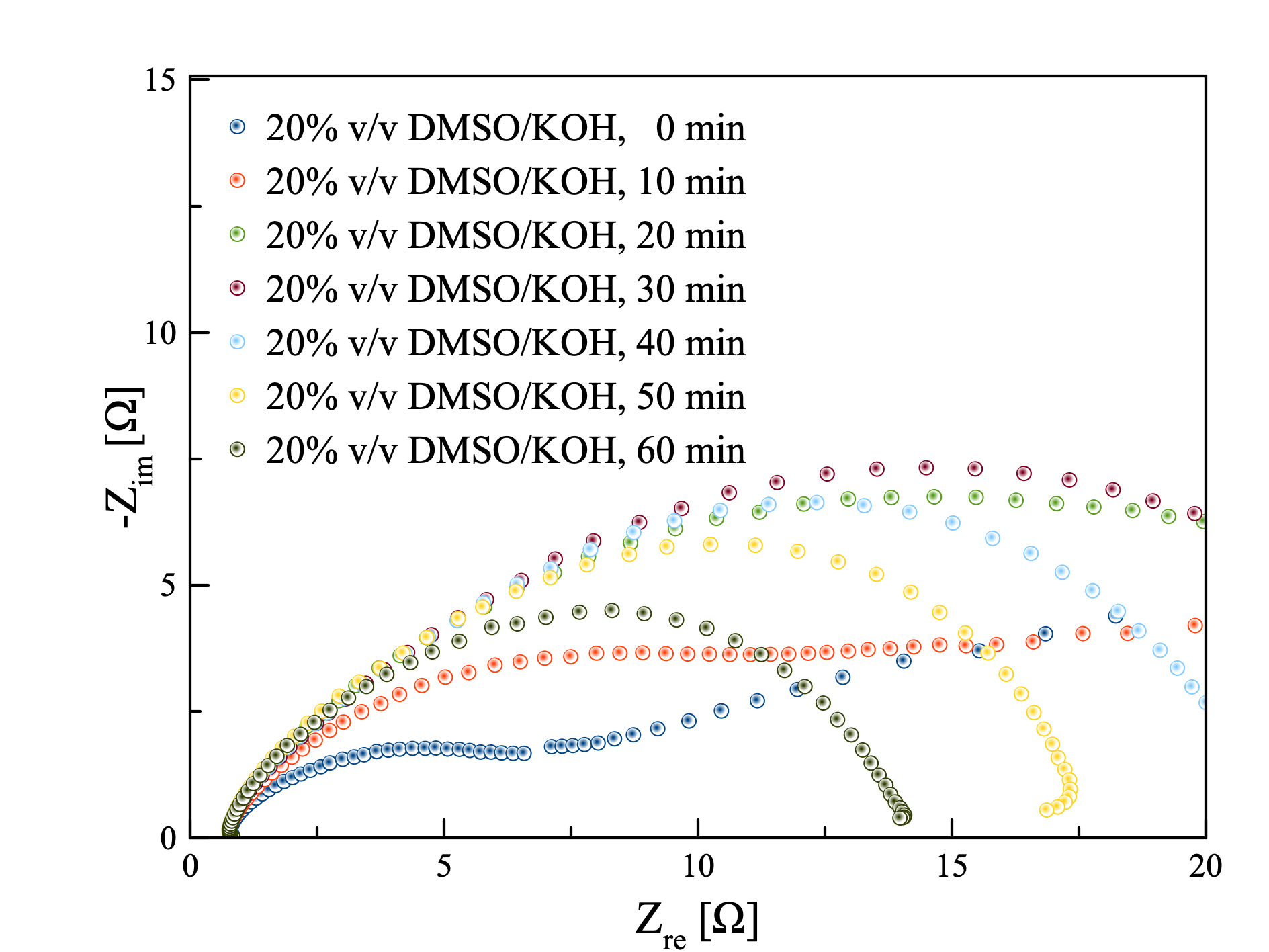


**Figure S5.** Nyquist plot of 20% v/v DMSO/KOH electrolyte at different soaking times (0-60 min).
